# The Gut Microbiota–Host Epigenetic Axis: An Emerging Biological Framework for Understanding Ethnic Disparities in Type 2 Diabetes Mellitus Susceptibility

**DOI:** 10.3390/nu18162668

**Published:** 2026-08-15

**Authors:** Mohamed Zaiou

**Affiliations:** Institut Jean-Lamour, Université de Lorraine, UMR 7198 CNRS, 54505 Vandoeuvre-les-Nancy, France; mohamed.zaiou@univ-lorraine.fr

**Keywords:** epigenetics, exposome, health disparities, insulin resistance, microbiome

## Abstract

Persistent racial and ethnic disparities in type 2 diabetes mellitus (T2DM) are incompletely explained by genetic susceptibility, obesity, lifestyle, and socioeconomic factors, suggesting that additional biological mechanisms may link environmental exposures to metabolic disease risk. Increasing evidence indicates that the exposome, encompassing environmental exposures across the life course, may influence long-term metabolic health through molecular mechanisms that integrate environmental signals with host biology. This Review synthesizes epidemiological, experimental, and mechanistic evidence on the gut microbiota–host epigenetic axis and its potential role in linking environmental exposures to population differences in T2DM susceptibility. Gut microbial dysbiosis alters the production and metabolism of short-chain fatty acids, bile acids, and tryptophan-derived metabolites. These microbial metabolites can modulate host signaling and epigenetic processes, including DNA methylation, histone modifications, and non-coding RNA activity, which may influence the expression of genes involved in glucose homeostasis, inflammation, and insulin sensitivity. Conversely, host epigenetic programs may influence intestinal barrier integrity and immune responses, thereby potentially affecting the gut microbial ecosystem, highlighting the reciprocal nature of host–microbiota interactions. We further examine how diet, psychosocial stress, environmental pollutants, and socioeconomic conditions may shape the microbiota–epigenetic axis across diverse populations, providing a framework for understanding differences in metabolic susceptibility without attributing disparities to intrinsic biological variation. However, direct human evidence linking the gut microbiota–host epigenetic axis to racial and ethnic disparities in T2DM risk remains limited, and much of the current framework is based on mechanistic studies, animal models, and associative human data rather than direct causal evidence. Further research in diverse human populations is needed to validate these pathways and establish their contribution to T2DM disparities. This framework may inform biomarker discovery, stratification, precision nutrition, and microbiome-targeted interventions and generate hypotheses for equitable prevention and personalized management of T2DM across diverse populations.

## 1. Introduction

Type 2 diabetes mellitus (T2DM), characterized by insulin resistance (IR) and progressive pancreatic β-cell dysfunction, affects more than 800 million adults worldwide [1]. Despite its global prevalence, the burden of T2DM is not equally distributed across populations. Racial and ethnic populations experience disproportionate burden of T2DM risk, including earlier disease onset and a greater burden of complications and mortality [2,3]. These differences in disease burden are also reflected in higher rates of macrovascular complications, including coronary heart disease and stroke, which are major contributors to diabetes-related mortality [3,4]. Although traditional risk factors, including obesity, physical inactivity, and genetic predisposition, are associated with T2DM development, they do not fully explain persistent interethnic differences in disease burden. Increasing evidence indicates that these disparities may be related to structural and environmental determinants that are reflected in the exposome, defined as the cumulative lifetime burden of environmental exposures, including diet, pollutants, psychosocial stress, medications, and socioeconomic adversity [3]. However, the pathways through which these external exposures may be associated with long-term metabolic dysfunction remain incompletely understood. Addressing this gap requires identification of molecular systems capable of translating environmental signals into durable biological responses. Two interconnected biological systems have emerged as potentially associated with this process: the host epigenome and the gut microbiome.

The gut microbiome is increasingly recognized as an important component of metabolic homeostasis. Alterations in microbial composition and function have consistently been associated with chronic low-grade inflammation, IR, and impaired glucose regulation in T2DM [5]. In parallel, epigenetic mechanisms, including DNA methylation (DNAmet), histone modifications, and non-coding RNAs (ncRNAs), provide dynamic interfaces that may link environmental, nutritional, and psychosocial exposures to changes in gene expression without altering the DNA sequence [6]. Importantly, both gut microbiota composition and epigenetic signatures vary across populations, which may reflect complex interactions among environmental exposures [7,8]. Recent evidence indicates that microbial metabolites, including short-chain fatty acids (SCFAs), secondary bile acids, and tryptophan-derived compounds, are involved in host epigenetic regulation, while host epigenetic programs may influence intestinal barrier integrity, immune responses, and microbial ecology. Together, these reciprocal interactions are conceptualized as the gut microbiota–epigenome axis, providing a mechanistic framework through which environmental exposures may be linked to biological processes relevant to metabolic health and variation in T2DM susceptibility across populations. In this review, we synthesize current evidence on the interactions between the gut microbiome, epigenetic regulation, and environmental exposures in T2DM, with a particular focus on their potential role in understanding some of these racial and ethnic disparities. We highlight microbial metabolites, epigenetic mechanisms, and the gut microbiota–host epigenetic axis as an integrated biological system that may constitute a mechanistic bridge linking environmental exposures to population differences in T2DM susceptibility. A deeper mechanistic understanding of these interactions may support the development of more precise and equitable strategies for the prevention and treatment of T2DM across populations. Before discussing the molecular mechanisms underlying this framework, it is important to examine the epidemiological evidence documenting persistent racial and ethnic disparities in T2DM. While direct causal evidence in human multinational and multi-ethnic cohorts remains an ongoing area of investigation, this Review integrates established observational, animal, and mechanistic multi-omics data to establish a plausible biological hypothesis linking exposome-related factors to population-level metabolic risk.

## 2. Ethnic Disparities in T2DM: Epidemiological Patterns and the Biological Impact of Social and Environmental Exposures

### 2.1. Global Epidemiological Evidence and Patterns of Ethnic Disparities

Ethnicity refers to socially constructed categories based on shared cultural, historical, linguistic, and ancestral characteristics that shape social identity and lived experiences [9]. Throughout this review, we distinguish race and ethnicity, which are primarily social constructs, from genetic ancestry, which refers to inherited patterns of human genetic variation. Although these concepts may be correlated, they are not interchangeable. While ethnic classifications may correlate with ancestry-related genetic variation, contemporary epidemiological evidence indicates that ethnicity itself is not an intrinsic biological determinant of disease risk. Rather, observed differences in T2DM risk arise from complex interactions among genetic susceptibility, environmental exposures, socioeconomic conditions, and structural determinants of health. Globally, T2DM prevalence varies substantially across ethnic and population groups, with disproportionately high burdens observed in several minority, Indigenous, and migrant populations, even after adjustment for traditional metabolic risk factors such as body mass index (BMI), lifestyle behaviors, and socioeconomic status. Indigenous populations, including American Indian and Alaska Native communities, experience some of the highest reported rates of T2DM, accompanied by early disease onset and an increased risk of cardiovascular and renal complications [10,11]. Similarly, populations of African ancestry in the United States and Europe exhibit a higher prevalence and earlier onset of T2DM compared with European-origin populations, along with a disproportionate burden of cardiometabolic complications [12,13,14]. Hispanic/Latino populations also demonstrate elevated prevalence and incidence, with marked heterogeneity across subgroups [15]. In Asian populations, including South Asian and East Asian groups, T2DM often develops at younger ages and at lower BMI levels compared with White populations, reflecting population-specific metabolic phenotypes that may include increased visceral adiposity and reduced lean mass (the “thin-fat” phenotype) [16,17,18,19,20,21]. In South Asian populations specifically, diabetes risk is approximately twofold higher at BMI levels around 22–25 kg/m^2^, well below conventional BMI thresholds for overweight and obesity in European populations [18,21]. Native Hawaiian and Pacific Islander populations similarly exhibit high and rising rates of T2DM and related complications [22]. In European settings, migrant populations originating from South Asia, Africa, and the Middle East experience a two- to fivefold higher risk of T2DM compared with host populations, with persistent disparities remaining even after adjustment for conventional risk factors [23,24].

Collectively, these observations suggest that ethnic disparities in T2DM are not necessarily attributable to population-intrinsic biology but emerge from reproducible interactions between environmental exposures, developmental trajectories, and structural determinants of health.

### 2.2. Limits of Traditional Risk Factors and the Need for Biological Embedding Frameworks

The epidemiological patterns described above highlight the need for mechanistic frameworks capable of explaining how environmental and social exposures translate into long-term metabolic susceptibility. While the “thrifty genotype” hypothesis has historically been proposed to account for population differences in diabetes susceptibility, contemporary evidence suggests that genetic predisposition alone is insufficient to fully explain the magnitude and persistence of ethnic disparities [22,25,26,27]. Consequently, increasing attention has focused on biological embedding frameworks. These models describe how structural, political, and economic inequities actively shape macro-level exposures that literally penetrate “under the skin and into the gut” throughout the life course, becoming incorporated into molecular and physiological processes that ultimately influence T2DM risk [28,29].

### 2.3. Biological Embedding of Social and Environmental Exposures

Applying this framework to T2DM highlights how structural inequalities, including residential segregation and unequal access to healthy food environments, may be associated with differences in dietary patterns and sustained metabolic risk in marginalized populations [30]. Neighborhood deprivation further limits opportunities for physical activity while amplifying contact with environmental pollutants and chronic psychosocial stressors [31]. Chronic activation of stress-response systems, particularly the hypothalamic–pituitary–adrenal (HPA) axis, represents one potential pathway through which social adversity may be translated into metabolic dysfunction. Sustained HPA axis dysregulation has been associated with IR, visceral adiposity, and impaired glucose metabolism, although the precise downstream molecular mediators remain incompletely defined [32].

The exposome framework provides a comprehensive model for capturing the cumulative burden of environmental exposures across the life course. However, the molecular mechanisms through which these exposures become integrated into host biology and contribute to persistent metabolic dysfunction, and ultimately to ethnic differences in T2DM susceptibility, remain incompletely understood. Increasing evidence suggests that this process involves environmentally responsive biological systems capable of translating external exposures into long-lasting changes in host physiology.

Among the biological systems implicated in this process, the gut microbiota (GM) and the epigenome have emerged as highly dynamic and environmentally responsive systems that may be involved in patterns of long-term metabolic adaptation. The following sections summarize current evidence linking GM dysbiosis to T2DM before examining its interactions with epigenetic regulation.

## 3. Gut Microbiota Dysbiosis in T2DM

The GM has emerged as a central regulator of host metabolism and immune homeostasis, making it a plausible mediator translating environmental exposures into biological outcomes. GM dysbiosis, defined as disruption of the composition and function of a healthy microbial ecosystem, has been consistently associated with obesity, chronic low-grade inflammation, IR, and T2DM. These observations suggest that the gut microbiome may contribute to metabolic dysfunction through functional changes extending beyond simple bacterial counts [33].

### 3.1. Core Features of Dysbiosis in T2DM

In T2DM, GM dysbiosis is generally characterized by reduced microbial diversity, the depletion of beneficial commensal bacteria and an enrichment of opportunistic or pro-inflammatory taxa. A recurrent finding across metagenomic studies is the reduction in butyrate-producing bacteria, including *Faecalibacterium prausnitzii*, *Roseburia* spp., and *Eubacterium rectale*, alongside an increased abundance of pro-inflammatory taxa such as *Escherichia coli* and specific *Clostridium* species [5,34,35,36]. These compositional changes are associated with reduced production of SCFAs, particularly butyrate, which plays a central role in maintaining intestinal barrier integrity, regulating immune responses, and modulating host metabolic pathways [37]. Dysbiosis is also associated with altered bile acid metabolism, increased branched-chain amino acids (BCAAs), and changes in tryptophan-derived metabolites, all of which may contribute to impaired glucose homeostasis and IR.

### 3.2. Functional Convergence Across Populations

Although specific microbial taxa associated with T2DM vary across studies and populations, largely due to differences in diet, geography, medication use, and methodological factors, a more consistent pattern emerges at the functional level. Across diverse cohorts, T2DM is associated with convergent alterations in microbial metabolic pathways, including reduced SCFA biosynthesis, dysregulated bile acid metabolism, and altered pathways related to endotoxin production and signaling [35,38,39]. Accordingly, functional convergence may provide a more robust mechanistic framework than taxonomic composition for understanding GM alterations across ethnically diverse populations with T2DM. Importantly, pharmacological treatment, most notably metformin, represents a major confounding factor in human microbiome studies. Metformin substantially alters gut microbial composition and metabolite profiles (e.g., increasing *Escherichia* species and altering bile acid pools), making it essential to distinguish drug-induced alterations from core T2DM-associated dysbiosis.

### 3.3. Mechanistic Links to Insulin Resistance

GM dysbiosis has been consistently associated with IR through multiple interconnected pathways [39,40]. Reduced SCFA availability may compromise intestinal barrier integrity and may be associated with increased permeability and the translocation of lipopolysaccharides (LPS) into the circulation. This metabolic endotoxemia may activate Toll-like receptor 4 (TLR4)-dependent inflammatory signaling pathways, including c-Jun N-terminal kinase (JNK) and inhibitor of nuclear factor kappa-B kinase (IKK), which may contribute to impaired insulin signaling and reduced glucose uptake in peripheral tissues [41]. In parallel, dysbiosis-associated alterations in bile acid metabolism and gut hormone signaling influence incretin secretion, including glucagon-like peptide-1 (GLP-1), thereby further disrupting glucose homeostasis [42]. Together, these mechanisms highlight the potential relevance of the GM to systemic metabolic function and its association with molecular pathways involved in long-term metabolic adaptation.

### 3.4. Ethnic Variation and Environmental Sensitivity

As an environmentally responsive ecosystem, the GM is highly sensitive to dietary, environmental, and socioeconomic conditions that differ across populations and may contribute to interethnic variation in microbial composition. While some taxon-level differences exist between ethnic groups, functional metabolic disturbances, particularly reduced SCFA production and altered inflammatory signaling, are more consistently observed across populations. These observations suggest that ethnic variation in T2DM-associated microbiota may reflect environmentally shaped microbial profiles rather than fixed population-specific traits. Thus, understanding how environmental and socioeconomic conditions influence gut microbiota composition and function across ethnic groups is critical for elucidating the biological mechanisms that may contribute to population disparities in T2DM.

### 3.5. Conceptual Implication: Toward an Integrated Regulatory Axis

Beyond its role in metabolic regulation, the GM represents a key interface between environmental exposures and host physiology. Microbial metabolites such as SCFAs, bile acids, and tryptophan derivatives can influence metabolic pathways and act as signaling molecules capable of modulating host gene expression. These properties position the GM not merely as a metabolic organ, but as a dynamic interface through which environmental exposures can influence host gene regulation. By generating bioactive metabolites capable of modulating epigenetic processes, the GM provides a mechanistic foundation for the gut microbiota–epigenome axis, an integrated regulatory system increasingly investigated in relation to T2DM pathogenesis. Importantly, although these functional alterations in microbial metabolism represent common pathophysiological pathways in T2DM, baseline microbiome composition and functional capacity, as well as their response to environmental perturbations, may vary across populations owing to differences in dietary patterns, environmental exposures, lifestyle, and socioeconomic conditions. Consequently, the gut microbiota–epigenome axis could provide a mechanistic framework for understanding how differentially patterned exposomal profiles may contribute to ethnic disparities in T2DM susceptibility.

## 4. Epigenetic Mechanisms in T2DM

Developing further this microbiota-derived signaling framework, epigenetic regulation represents a major molecular mechanism through which microbial metabolites may exert long-term effects on host gene expression relevant to T2DM. Unlike genetic mutations, epigenetic modifications are dynamic and potentially reversible, making them important mediators through which chronic overnutrition, physical inactivity, aging, and psychosocial stress may contribute to IR and β-cell dysfunction, as demonstrated in experimental and observational studies. Epigenetic regulation in T2DM operates through three interconnected functional layers: (i) DNAmet as a relatively stable molecular record that may reflect environmental exposure, (ii) histone and chromatin modifications as dynamic regulators of transcriptional accessibility, and (iii) ncRNAs as regulators of gene expression at both transcriptional and post-transcriptional levels. These mechanisms differ in their stability, reversibility, and regulatory functions, yet they operate in a coordinated manner to shape metabolic gene expression and cellular adaptation.

### 4.1. DNA Methylation and Metabolic Dysregulation

DNAmet is one of the most extensively studied epigenetic mechanisms in T2DM and involves the addition of methyl groups to cytosine residues, which can alter gene transcription depending on genomic context and regulatory location. Epigenome-wide association studies (EWAS) have identified differential DNA methylation pattern in metabolic tissues including pancreatic islets, skeletal muscle, adipose tissue, and peripheral blood, affecting genes involved in insulin signaling, glucose transport, and mitochondrial function [43,44,45]. Altered methylation of key metabolic regulator genes, such as *PPARGC1A*, *TCF7L2*, and the thioredoxin-interacting protein gene (*TXNIP*), has been associated with impaired oxidative metabolism, reduced insulin secretion, and increased oxidative stress. Importantly, several of these epigenetic signatures are detectable in peripheral blood and have been associated with subsequent risk of incident T2DM or hyperglycemia, supporting their potential utility as early biomarkers of metabolic dysfunction [44,46].

Multi-ethnic studies further indicate that DNAmet patterns associated with metabolic traits are not fully conserved across populations. Population-specific differentially methylated loci have been identified in cohorts of African ancestry [47], while other studies have identified both shared and population-specific methylation patterns [48]. These findings suggest that genetic ancestry and environmental and socio-environmental factors may jointly contribute to differences in epigenetic signatures across diverse populations. Such evidence underscores the importance of considering ethnic and environmental context when interpreting epigenetic biomarkers and support the hypothesis that differentially patterned exposomal influences may contribute to heterogeneity in T2DM risk.

### 4.2. Histone Modifications and Chromatin Regulation

Histone modifications regulate chromatin accessibility and thereby control the transcriptional activity of genes involved in metabolic and inflammatory pathways. In insulin-resistant states, dysregulated histone acetylation and methylation patterns have been observed in hepatic, adipose, and skeletal muscle tissues and are associated with chronic low-grade inflammation and impaired glucose homeostasis [49,50]. In particular, an imbalance between histone acetyltransferases (HATs) and histone deacetylases (HDACs) has been associated with differences in the expression of key metabolic targets, such as the glucose transporter gene *SLC2A4* (encoding GLUT4) and *TXNIP*, which may be relevant to peripheral glucose uptake and β-cell survival [50]. These chromatin-level changes are influenced by environmental factors, including diet, chronic hyperglycemia, and oxidative stress, and many histone modifications are dynamic and potentially reversible, highlighting their potential therapeutic relevance. Population-based epigenomic studies suggest that histone modification patterns may also vary across ethnic groups, reflecting differences in environmental exposures, socioeconomic conditions, and aging-related processes, although human data in this domain remain strictly very limited.

### 4.3. ncRNAs and Post-Transcriptional Regulation

ncRNAs, including microRNAs (miRNAs), long ncRNAs (lncRNAs), and circular RNAs (circRNAs), are closely associated with the regulation of metabolic pathways at the post-transcriptional level in T2DM [51,52]. Specifically, altered expression of miR-103/107 and miR-29 has been associated with impaired peripheral insulin sensitivity through effects on components of insulin signaling, while miR-375 has been implicated in regulating pancreatic β-cell survival and insulin exocytosis. Concurrently, lncRNAs such as *MALAT1* and *GAS5* have been proposed to participate in metabolic stress responses; under diabetic glucotoxicity, their dysregulation may alter hepatic gluconeogenic pathways [53].

These regulatory RNAs operate within complex competing endogenous RNA (ceRNA) networks and can be packaged into circulating extracellular vesicles, which may contribute to their stability and detectability in peripheral blood [52]. As such, ncRNAs represent important regulators of metabolic processes and potential non-invasive biomarkers of disease progression. Emerging evidence suggests that ncRNA expression profiles may differ among populations in response to distinct environmental exposures and metabolic contexts, potentially contributing to interindividual variation in metabolic susceptibility, although direct evidence linking these differences to ethnic disparities in T2DM remains limited [51,52]. Collectively, these findings suggest that ncRNA-mediated regulation is influenced not only by metabolic dysfunction but also by environmental and social context. Consequently, variation in ncRNA expression across ethnic populations may represent one potential mechanism through which differential lifetime exposures could contribute to differences T2DM susceptibility and progression.

## 5. The Gut Microbiota–Host Epigenome Axis as a Mediator of Metabolic Susceptibility

Having established the independent associations of microbial dysbiosis and host epigenetic dysregulation in T2DM, these two dynamic systems may be considered as interconnected components of a broader biological network. Crucially, this bidirectional gut microbiota–host epigenetic axis may provide a mechanistic framework through which population-specific exposomal factors may contribute to ethnic disparities in T2DM susceptibility. Microbial metabolites, including short-chain fatty acids (SCFAs), secondary bile acids, and tryptophan-derived metabolites, can modulate DNA methylation, histone modifications, and non-coding RNA (ncRNA) activity. These epigenetic changes may influence the expression of genes involved in inflammation, insulin sensitivity, and glucose metabolism [54,55,56]. Conversely, host epigenetic regulation of intestinal barrier integrity, immune responses, and metabolic signaling is associated with differences in gut microbiota composition and functional activity, supporting the possibility of a dynamic reciprocal relationship between the gut and microbial ecosystem [54,55]. This integrated regulatory network provides a biological framework through which dietary, lifestyle, environmental, and genetic factors may interact to influence metabolic phenotypes. The dysregulation of this axis has been linked to obesity, IR, metabolic dysfunction-associated steatotic liver disease (MASLD), and T2DM, suggesting that dysregulation of this axis may contribute to interindividual and population-level differences in metabolic disease susceptibility [51,54,55,56].

In this context, investigating how ethnicity-related environmental exposures interact with gut microbiota composition and function, together with host epigenetic regulation, may provide insights into mechanisms underlying population differences in T2DM susceptibility. Nevertheless, direct causal evidence linking the gut microbiota–epigenome axis specifically to ethnic disparities in T2DM remains limited and is currently supported primarily by observational, animal, and cross-sectional studies [51]. Rather than asserting definitive causality in humans, this framework serves as a biologically plausible working model that bridges established animal-model mechanics with emerging human exposome data, highlighting the urgent need for prospective, multi-omic causal mediation studies in diverse populations.

The subsequent sections examine (i) how gut microbiota-derived metabolites regulate host epigenetic mechanisms, (ii) how host epigenetic processes reshape the intestinal ecosystem through reciprocal feedback, and (iii) how this bidirectional axis may contribute to ethnic variation in T2DM susceptibility.

### 5.1. Gut Microbiota-Mediated Host Epigenetic Modifications

The GM is a metabolically active ecosystem associated with the production of a wide range of bioactive metabolites such as SCFAs, bile acids, and metabolites derived from amino acids. These substances have been implicated in epigenetic regulatory processes in the host and in the maintenance of metabolic homeostasis [57,58,59,60]. There is a growing recognition of these metabolites as crucial mediators in the communication between the host and microbes, although direct causal evidence in human populations is still scarce. It is important to emphasize that much of the detailed mechanistic evidence describing these metabolite–epigenome interactions is derived from animal or in vitro models. While these models establish biological plausibility, caution is warranted when extrapolating preclinical findings to human population disparities.

Through these metabolites, the GM may be involved in various host epigenetic mechanisms, which may be associated with changes in gene networks that are crucial for glucose metabolism, insulin sensitivity, mitochondrial function, and immune responses relevant to the pathogenesis of T2DM [58,60]. Among these metabolites, SCFAs are recognized as among the most extensively studied mechanistic links between microbial activity and host epigenetic regulation [58,59,60]. The subsequent sections outline the key epigenetic mechanisms by which metabolites derived from the microbiota affect the metabolic functions of the host in T2DM.

#### 5.1.1. SCFAs and Histone Regulation

Histone acetylation represents one of the best-characterized epigenetic mechanisms potentially linking gut microbiota-derived metabolites with the regulation of host gene expression. SCFAs, particularly butyrate, have been reported to act as endogenous inhibitors of HDACs, which may favor a more open chromatin state and be associated with changes in the transcription of genes involved in insulin sensitivity and anti-inflammatory signaling [61]. In addition to inhibiting HDACs, propionate and butyrate have been found to directly stimulate the histone acetyltransferase (HAT) p300 through their respective acyl-CoAs [62]. The potential in vivo role of SCFAs in modulating histone acetylation has been demonstrated in germ-free mice, where the administration of microbiota-derived acetate, propionate, and butyrate was associated with increased tissue-specific acetylation of histones H3 and H4 [63]. Furthermore, acetate may contribute to epigenetic regulation by acting as a precursor for acetyl-CoA, which aids in HAT-mediated chromatin remodeling. An increase in intracellular acetyl-CoA, which serves as a substrate for HATs, may thus facilitating histone acetylation [64]. In summary, HDAC inhibition by butyrate, together with enhanced acetyl-CoA availability, has been associated with differences in gene expression of key metabolic regulators such as PGC-1α and GLUT4, which in turn may improve mitochondrial function and glucose uptake [65,66].

In the context of T2DM-related gut dysbiosis, a diminished presence of SCFA-producing bacterial genera, such as *Faecalibacterium* and *Roseburia*, correlates with a reduction in SCFA availability [58,60]. This decline is linked to compromised intestinal barrier integrity, chronic low-grade systemic inflammation, and reduced metabolic flexibility. The scarcity of SCFAs may lead to decreased histone acetylation and modifications in the transcription of genes that are crucial for insulin signaling and β-cell functionality, while simultaneously promoting the activation of pro-inflammatory pathways, including NF-κB signaling [58,61,65,66].

Recent studies suggest that interactions between the GM and the host epigenome are not solely dependent on SCFAs. Global histone acetylation patterns can also be influenced by tissue-specific, SCFA-independent mechanisms, highlighting additional layers of epigenetic regulation [67]. Collectively, these observations support a potential mechanistic link among gut microbial metabolism, histone acetylation, transcriptional regulation, IR, and metabolic dysfunction relevant to T2DM.

#### 5.1.2. Microbial Metabolites and DNA Methylation in T2DM

In addition to their associations with histone modifications, GM-derived metabolites may also be associated with differences in host DNAmet by modulating one-carbon metabolism, methyl-donor availability, and DNA methyltransferase (DNMT) activity. Among SCFAs, butyrate has been reported to modulate the expression and activity of DNMTs, including DNMT1 and DNMT3B, thereby influencing DNA methylation patterns and the epigenetic landscape of key metabolic genes [68,69,70]. Mechanistic studies in human cell models have demonstrated that butyrate-mediated HDAC inhibition can accelerate the mRNA decay of *DNMT3B* [69] and promote the ubiquitin-dependent proteasomal degradation of DNMT1 protein [70], highlighting a potential crosstalk pathway linking SCFA availability to the machinery of DNA methylation.

Propionate has been suggested to affect DNAmet by altering the metabolism of methyl donors, influencing the availability of intracellular S-adenosylmethionine (SAM), and impacting chromatin-associated signaling pathways, although the precise mechanisms are not fully elucidated [71,72]. Furthermore, reductions in folate-producing bacteria associated with dysbiosis may lead to decreased availability of methyl donors and thereby hinder SAM synthesis, which could potentially result in abnormal DNA methylation patterns [73,74]. Supporting this notion, Guo et al. illustrated that SCFAs derived from GM may affect DNAmet patterns linked to an increased risk of T2DM in individuals predisposed to obesity. This provides experimental support for a microbiota–epigenome–metabolism relationship, rather than confirming a direct causal link between SCFAs and the onset of diabetes [75]. In line with these mechanistic findings, EWAS and candidate-gene analyses conducted on peripheral blood, skeletal muscle, adipose tissue, and pancreatic islets from T2DM patients have revealed changes in DNAmet of genes such as *TXNIP*, *ABCG1*, *PPARGC1A*, and *TCF7L2*, which are involved in the regulation of insulin secretion, glucose metabolism, and mitochondrial function [76,77,78,79]. Collectively, these findings suggest that metabolites derived from the GM may modify the host epigenetic landscape through various mechanisms, including histone modifications and DNA methylation, which in turn may influence the expression of metabolic genes and play a role in the pathogenesis of T2DM. Although this section has concentrated on the epigenetic regulation driven by the microbiota, it is important to note that the host epigenome also significantly influences the intestinal environment and microbial ecology, highlighting a reciprocal relationship that will be discussed further below.

### 5.2. Host Epigenetic Regulation of Gut Microbiota Composition

While it is well established that bacterial metabolites can influence host genetics, the reverse is also plausible: the host’s epigenetic regulatory mechanisms may be associated with differences in the gut ecosystem. In T2DM, chronic hyperglycemia, IR, and inflammation are associated with extensive epigenetic remodeling across intestinal epithelial, immune, and metabolic tissues. These host-associated alterations, including DNAmet, histone modifications, and ncRNAs, may continue to change in the ecological and biochemical environment of the gut. In T2DM, disruptions in these regulatory pathways are associated with differences in gene expression patterns relevant to intestinal homeostasis and the structure of the microbial community. The subsequent sections will examine the specific mechanisms associated with this proposed host-to-microbial regulatory relationship.

#### 5.2.1. Luminal miRNAs and Microbial Gene Regulation

Host-derived miRNAs are continuously produced by intestinal epithelial and immune cells, encapsulated within protective extracellular vesicles, and released into the intestinal lumen. Here, they can be actively taken up by gut bacteria, potentially affecting microbial gene expression and growth [80,81,82]. Liu et al. experimentally validated this cross-kingdom regulatory mechanism, demonstrating that host luminal miRNAs directly target bacterial transcripts and can thereby modulate microbial replication [80]. Although these interactions may represent a distinct control loop between the host and microbiota, their overall extent and functional importance in human T2DM remain to be fully clarified. In T2DM, metabolic stress associated with persistent hyperglycemia and inflammation may be associated with changes in the epigenetic regulation and transcriptomic profiles of epithelial and immune miRNAs, potentially contributing to alterations in the composition of the luminal miRNA pool.

According to the synthesis by Cuinat et al. [81], these host-associated alterations are linked to differences in the homeostatic regulation of microbial populations. Such changes may also coincide with differences in the stability and composition of microbial communities, resulting in a reduced presence of beneficial taxa like *Akkermansia muciniphila* and *Faecalibacterium prausnitzii*, while potentially allowing for the proliferation of opportunistic pathobionts.

Ultimately, as noted by Liang et al. [82] in relation to chronic inflammatory states, these dysregulated ncRNAs may contribute to maladaptive cross-kingdom interactions. Consequently, luminal miRNAs have emerged as a potentially relevant molecular component of the relationship between host epigenetic state, microbial gene regulation, and population dynamics in T2DM.

#### 5.2.2. Epigenetic Regulation of Intestinal Barrier Integrity

In T2DM, ongoing hyperglycemia and systemic inflammation contribute to significant epigenetic changes in intestinal epithelial cells [60]. These host-associated alterations may be linked to differences in the transcriptional regulation of genes relevant to maintaining epithelial integrity, resulting in a marked downregulation of crucial tight-junction proteins, including Claudin-1 (*CLDN1*), Occludin (*OCLN*), and Zonula Occludens-1 (*TJP1*) [60,83].

Disrupted barrier integrity is characterized by increased intestinal permeability, often referred to as “leaky gut”. This altered barrier state may be related to differences in the physicochemical environment within the lumen, including oxygen diffusion, nutrient gradients, and epithelial surface exposure [84]. These changes may be associated with shifts in the microbial habitat and the relative abundance of facultative anaerobes and pathobionts that thrive in oxygen-rich or inflamed conditions, while imposing a significant survival disadvantage on strict, fiber-fermenting anaerobic commensals.

Furthermore, barrier dysfunction may be associated with microbial invasion and the movement of immunogenic bacterial elements, including LPS, into the lamina propria, significantly amplifying both local mucosal and systemic inflammatory signaling [60,84]. This intensified inflammatory milieu may be associated with further epithelial epigenetic dysregulation, potentially coinciding with changes in microbial community structure.

#### 5.2.3. Remodeling of the Gut Metabolic Niche

Host epigenetic mechanisms are associated with gene expression programs relevant to the intestinal metabolic niche. Under diabetic metabolic stress, these synchronized host-side alterations may be associated with changes in epithelial barrier function, mucin production, mucus layer structure, and the secretion of mucosal antimicrobial peptides. Simultaneously, these epigenetic changes may be associated with differences in bile acid homeostasis and the activity of related transport and metabolic pathways, such as CYP7A1, the Farnesoid X Receptor (FXR), and the Takeda G-protein-coupled receptor 5 (TGR5) [56,85,86].

These processes may be associated with differences in the intestinal ecological niche, including variations in luminal oxygen gradients, nutrient availability, mucus composition, and immune selection pressures. These environmental differences may also be associated with shifts in microbial community composition, including differences in the relative abundance of obligate anaerobic commensals and inflammatory pathobionts, as reflected by the observed decrease in SCFA-producing taxa such as *Faecalibacterium prausnitzii* and *Roseburia* spp. [34,55].

As a result, microbial metabolic activity may shift from the production of beneficial metabolites such as butyrate to the formation of metabolites associated with metabolic dysfunction, including imidazole propionate, BCAAs, and compounds derived from trimethylamine (TMA). These metabolic alterations may be linked to impaired host insulin signaling and may promote systemic inflammatory responses, establishing a detrimental feedback loop that may contribute to progressive metabolic dysfunction [56,87,88]. In conclusion, host epigenetic dysregulation may contribute to the restructuring of the intestinal microenvironment, which may influence both microbial community composition and pathological metabolic outcomes.

### 5.3. The Gut Microbiota–Host Epigenetic Axis in T2DM and Racial Disparities

The gut microbiota–host epigenetic axis constitutes a dynamic regulatory interface through which environmental signals are integrated into host biology. Despite this shared architecture, substantial variation exists in T2DM susceptibility and metabolic outcomes across individuals and populations. Such heterogeneity may arise from differences in cumulative environmental exposures, host genetic background, and developmental history that shape the composition of the gut microbiota [89] and epigenetic regulation [90], supporting emerging models in which the microbiome may contribute to disparities in metabolic health.

Host genetic variation is associated with the baseline configuration of the microbiota-epigenetic axis, whereas environmental and cultural factors may be more strongly associated with its long-term functional variation. Indeed, individuals from diverse ethnic backgrounds living within the same geographical regions retain distinct microbial community structures that reflect dietary patterns, cultural practices, socioeconomic conditions, migration history, and lifestyle behaviors rather than genetic ancestry alone [91]. Similarly, EWAS have identified both shared and ancestry-associated DNA methylation signatures linked to T2DM onset, suggesting that host epigenetic landscapes may be associated with population-specific transcriptional contexts in which similar microbial stimuli may coincide with divergent metabolic outcomes [92]. Collectively, these findings indicate that while host genetic variation may contribute to the initial biological configuration of this axis, environmental exposures appear to be more closely related to its dynamic changes over time.

It is important to distinguish genetic ancestry from race or ethnicity. Although host genetics and genetic ancestry may influence the baseline configuration of the gut microbiota–host epigenetic axis, its regulation is highly dynamic and shaped by environmental, dietary, socioeconomic, and structural exposures across the life course. Accordingly, ethnic disparities in T2DM are best understood as arising from interactions between inherited biological variation and cumulative environmental and social influences rather than from immutable genetic differences. To avoid biological essentialism, ancestry-associated epigenetic variations should be interpreted primarily within the context of differential exposures across populations rather than as evidence of fixed biological divergence. The exposome, including diet, early-life microbial colonization, maternal nutrition, psychosocial stress, exposure to xenobiotics or antibiotics, migration, and urbanization, is strongly associated with variation in this regulatory system, particularly during critical developmental windows. In accordance with the Developmental Origins of Health and Disease (DOHaD) framework, many of these exposures have been associated with persistent microbial and epigenetic patterns established early in life, which may in turn be related to variation in metabolic health across the lifespan [89,90,93]. Migration studies further highlight the plasticity of this system, demonstrating that rapid transitions to Westernized environments are associated with substantial changes in GM composition and function within a single generation [94].

Multi-omics investigations further support this integrative framework by revealing consistent associations between microbial functional outputs, metabolomic profiles, and host epigenetic signatures related to glucose metabolism and IR across diverse populations [91,95]. Importantly, emerging evidence indicates that microbiota-derived metabolites are associated with environmental exposures and epigenetic patterns related to immune function, inflammatory pathways, and metabolic homeostasis [60,96,97]. Although much of the current evidence is associative, converging data from experimental models, longitudinal cohorts, and emerging multi-omics studies suggest that the gut microbiota–host epigenetic axis may be involved in the relationship between environmental exposures and metabolic health.

**Figure 1:**
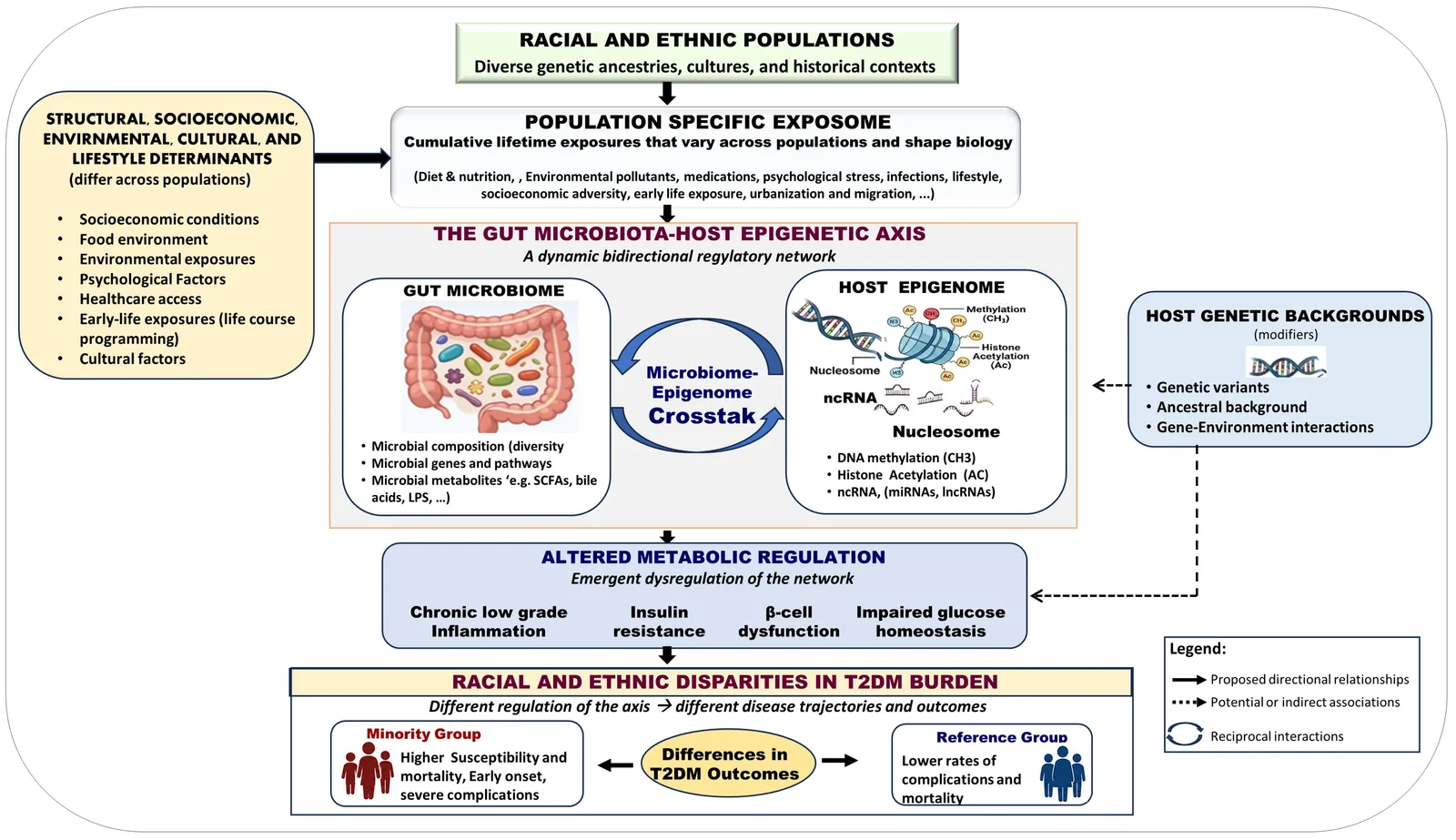
Conceptual framework illustrating how the exposome shapes the gut microbiota–host epigenetic axis and contributes to racial and ethnic disparities in T2DM.

From a systems biology perspective, the gut microbiota–host epigenetic axis does not converge toward a single universal metabolic state. Instead, each individual may exhibit a distinct regulatory baseline associated with lifelong interactions between inherited biological traits and cumulative environmental exposures. This baseline is likely to be associated with variation in the resilience of the system, its adaptive capacity under metabolic stress, and the threshold at which homeostatic regulation may become disrupted, potentially contributing to persistent IR and T2DM. Accordingly, ethnic disparities in T2DM should be understood as potentially emerging from differences in the regulation of a shared adaptive network influenced by the exposome rather than being attributed to intrinsic biological differences between populations [98]. This framework supports precision medicine approaches that integrate microbiome function, epigenetic regulation, and social determinants of health to improve metabolic outcomes across diverse populations [57,60,96]. Collectively, these findings highlight the gut microbiota–host epigenetic axis as a central interface through which environmental exposures may influence biological processes, potentially contributing to interindividual and population-level variation in T2DM susceptibility (Figure 1).

To summarize, the gut microbiota-epigenetic axis represents a key regulatory network associated with relationships among microbial ecology, host epigenetic mechanisms, and metabolic homeostasis. Its disruption may be associated with a pattern of co-occurring dysbiosis, epigenetic dysregulation, inflammation, IR, and β-cell dysfunction in T2DM. This integrated framework may help explain inter-individual and population-level variation in disease susceptibility. Overall, ethnic disparities in T2DM may be understood not as reflecting immutable biological differences, but rather as potentially arising from dynamic relationships among environmental factors, host biological diversity, and microbiota-epigenetic regulation throughout the lifespan. Future longitudinal multi-omics research that integrates microbiome, epigenome, metabolome, exposome, and clinical phenotyping will be vital for evaluating this model and informing precision prevention strategies.

## 6. Translational Implications: The Gut Microbiota–Epigenome Axis in Ethnic Disparities of T2DM

The growing understanding of interactions between the GM and the host epigenome has important implications for improving T2DM risk prediction, prevention, and treatment across diverse populations. This emerging framework provides opportunities to integrate social and environmental determinants of health with molecular mechanisms, thereby supporting a more comprehensive understanding of population differences in T2DM risk and progression. Social adversity and other structural determinants of health have been associated with molecular signatures related to long-term metabolic health [99,100]. Accordingly, integrating exposomal, microbial, and epigenetic information into risk assessment may improve the identification of individuals and populations at increased risk beyond conventional clinical and genetic factors.

### 6.1. Risk Stratification and Early Detection

Conventional risk assessment methods that rely on BMI thresholds do not adequately capture metabolic heterogeneity across populations. This is particularly evident in populations exhibiting “thin-fat” phenotypes, such as South Asians, among whom elevated metabolic risk has been observed at lower BMI (23 kg/m^2^ for overweight and 25 kg/m^2^ for obesity, compared to standard thresholds of 25 kg/m^2^ and 30 kg/m^2^, respectively) [18,101]. Integration of multi-omic biomarkers, including gut microbial composition, circulating SCFAs, and host epigenetic marks (e.g., DNAmet at *TXNIP*, *ABCG1*, *SOCS3*), may aid in the early identification of high-risk individuals before overt hyperglycemia develops [46]. Future risk prediction models integrating metagenomics, metabolomics, epigenomics, clinical characteristics, and SDOH may offer enhanced risk stratification for T2DM susceptibility across diverse populations. Additionally, ongoing monitoring of microbial metabolites and evolving epigenetic signatures may provide innovative biomarkers for assessing disease progression and treatment efficacy, although prospective validation is still required.

### 6.2. Therapeutic Targeting of Gut Microbiota–Epigenome Pathways

Current glucose-lowering treatments mainly focus on managing metabolic abnormalities that arise after the onset of the disease. In contrast, the modulation of the gut microbiota–epigenome axis may represent a potential therapeutic approach for pathways involved in inflammatory pathways, insulin sensitivity, and metabolic homeostasis. Microbial metabolites, particularly SCFAs, have been associated with host metabolic processes through mechanisms involving activation of G-protein-coupled receptors and inhibition of HDACs, with potential implications for chromatin accessibility and gene expression related to glucose metabolism and immune regulation [102,103]. The increased activity of HDAC3 observed in individuals with T2DM may further support the investigation of epigenetic modulation as a therapeutic strategy [102].

Recent findings from preclinical and oncology studies provide additional support for the microbiome’s role in modulating host chromatin states, indicating the potential for microbiota-targeted or epigenetically active adjunct therapies in metabolic diseases [104]. However, direct evidence for sustained microbiota-mediated epigenetic changes in humans remains limited. Moreover, much of the mechanistic evidence derives from in vitro studies and germ-free/axenic animal models, which do not fully recapitulate the complexity and interindividual variability of human populations. Consequently, interventions aimed at the microbiota, such as probiotics, prebiotics, synbiotics, engineered probiotics, postbiotics, and small-molecule HDAC-modulating agents, should be regarded as promising research avenues rather than established treatments [35]. A critical limitation is that a significant proportion of mechanistic insights relies on in vitro or germ-free animal models, which cannot fully capture the physiological complexity, environmental diversity, and interindividual variation observed in human clinical settings.

### 6.3. Precision Nutrition and Microbiome-Informed Interventions

Diet is one of the factors most strongly associated with variation in both gut microbial ecology and downstream epigenetic regulation. Fiber-rich dietary patterns are associated with higher abundances of SCFA-producing bacteria and higher levels of butyrate, propionate, and acetate, metabolites that may influence histone acetylation, DNAmet, intestinal barrier integrity, and systemic glucose homeostasis [95,105]. In contrast, Western dietary patterns, which are marked by high consumption of saturated fats and refined carbohydrates, are associated with lower microbial diversity, reduced SCFA production, and chronic low-grade inflammatory states.

Recent studies demonstrate substantial interindividual variability in postprandial glycemic responses that may be predicted by microbiome-based algorithms, providing support for the concept of personalized nutrition [106]. Accordingly, microbiome-informed dietary interventions may support more individualized nutritional recommendations based on an individual’s microbial profile, metabolic phenotype, dietary habits, and cumulative environmental exposures rather than ethnicity alone [105,106]. These strategies may be particularly relevant to groups displaying diverse metabolic vulnerabilities, even when their BMI is comparable.

### 6.4. Precision Prevention and Clinical Implementation

Beyond treatment, the gut microbiota–epigenome axis offers considerable potential for precision prevention. Integration of microbial, epigenetic, metabolic, clinical, and environmental information may enable identification of individuals who are most likely to benefit from targeted lifestyle interventions before substantial metabolic dysfunction develops. Importantly, ethnicity should not be considered a biological determinant for clinical decision-making but rather a contextual variable reflecting heterogeneous environmental, dietary, socioeconomic, and psychosocial exposures accumulated across the life course. Instead, ethnicity should be interpreted as a contextual variable reflecting the cumulative influence of environmental, social, and healthcare-related exposures that may shape disease risk and response to intervention. Incorporating SDOH into microbiome-epigenome-based prediction models may therefore support more context-sensitive prevention strategies while avoiding essentialist interpretations of race or ethnicity. Because epigenetic programming may be particularly responsive to environmental exposures during critical developmental periods, including pregnancy, early childhood, and adolescence, preventive interventions implemented during these windows may be associated with more favorable long-term metabolic outcomes than interventions initiated later in adulthood. This life-course approach further highlights the potential relevance of incorporating microbiome-focused nutritional strategies into early prevention initiatives.

### 6.5. Translational Relevance to Health Disparities

The microbiota–epigenome axis provides a potential mechanistic framework linking structural social determinants of health (SDOH), including systemic food insecurity, chronic psychosocial stress, environmental xenobiotic exposure, and neighborhood deprivation, to metabolic disease risk through associated systemic physiological alterations [95,107,108]. By integrating frameworks from the social epidemiology of the microbiome, particularly as articulated by Dowd and Renson, it becomes evident that the human microbiota serves as a dynamic and adaptable pathway that is associated with socioeconomic and environmental adversities [28]. Rather than treating the microbial ecosystem as a passive byproduct of individual dietary choices, contemporary paradigms advanced by Amato et al. suggest that structural, political, and economic inequities are associated with differences in macro-level exposures that may, in turn, contribute to biological disparities in the host gastrointestinal tract across the life course [29].

Consequently, these adverse external exposures are associated with alterations in gut microbiome ecology, including patterns of gut dysbiosis, systemic inflammation, and reduced levels of metabolically relevant SCFAs, while also being associated with differences in host epigenetic regulation that may contribute to persistent disparities in T2DM outcomes. Importantly, ethnicity-associated differences in GM and epigenetic signatures may largely reflect heterogeneous environmental and socioeconomic exposures rather than intrinsic, unchangeable biological differences between ancestral populations [101]. Furthermore, these disrupted pathways may extend across generations, as maternal exposomal stress during the critical “first three months” window is associated with differences in the neonatal pioneer microbiome and early-life epigenetic programming, which may contribute to greater metabolic risk in subsequent generations. These insights indicate that forthcoming prevention strategies should incorporate multi-faceted interventions that mitigate adverse SDOH alongside clinical therapies to potentially reduce the risk of adverse microbiome and epigenetic trajectories associated with metabolic disease.

### 6.6. Emerging Clinical Tools and Future Translation

Recent developments in multi-omics integration, encompassing metagenomics, metabolomics, and epigenomics, are facilitating the identification of composite biomarkers for the stratification of metabolic risk. Future applications may involve microbiome-based diagnostics, epigenetic risk panels, engineered probiotics, postbiotic therapies, and small-molecule HDAC-modulating agents [35]. However, despite the substantial conceptual promise, the clinical application of these approaches is still in its early stages due to the scarcity of longitudinal multi-ethnic cohorts, considerable interindividual variability, and the absence of robust interventional validation.

Therapeutic approaches targeting the gut microbiota–host epigenetic axis are evolving rapidly, yet significant translational obstacles persist. Fecal microbiota transplantation (FMT) has shown transient changes in peripheral insulin sensitivity in proof-of-concept studies in individuals with metabolic syndrome; however, observed outcomes have been inconsistent, and long-term effeciacy remains to be established [109,110]. Similarly, dietary interventions, prebiotics, probiotics, postbiotics, and emerging epigenetic therapies have shown potential metabolic effects, but their clinical utility is limited by interindividual variability, heterogeneous study designs, and the lack of standardized protocols and long-term validation [111,112]. Together, these findings highlight the conceptual promise of targeting the gut microbiota–host epigenetic axis while underscoring that its clinical translation remains at an early stage and requires rigorous longitudinal validation in diverse human populations before routine implementation. Table 1 summarizes complementary microbiome- and epigenome-based strategies that collectively may support the clinical translation of the gut microbiota–host epigenetic axis, together with their biological targets, potential clinical applications, and current limitations.

## 7. Limitations and Future Directions in Studying Racial and Ethnic Disparities in T2DM Susceptibility

The intersection of the gut microbiome, host epigenome, and macro-environmental exposures presents substantial methodological challenges while offering important opportunities to enhance our understanding and ultimately mitigate health disparities. Presently, research is impeded by a lack of diversity within study cohorts, poor integration of multi-omic datasets, and a deficiency in strong causal evidence. To overcome these limitations, the following aspects must be addressed: (i) Future research should focus on improving population stratification. Ethnic groups are frequently regarded as uniform populations, which neglects significant differences in genetic ancestry, admixture, migration history, socioeconomic status, cultural practices, diet, lifestyle, and early-life exposures. This simplification may limit biological resolution, obscure metabolic risk patterns specific to subgroups, and complicate the differentiation between genetic, environmental, developmental, and microbiome-related factors associated with T2DM. Specifically, future studies must employ rigorous analytical approaches (such as admixture mapping and mediation analysis) to disentangle the contributions of genetic ancestry from social and environmental determinants, ensuring that epigenomic signatures are not misattributed to inherent ancestral biology. Notably, diet, cultural practices, lifestyle changes associated with migration, and early-life microbial exposures can influence the gut microbiome and epigenetic programming independently of genetic ancestry. By incorporating genetic, environmental, socioeconomic, cultural, and developmental elements into study design, researchers will be able to better reflect population diversity and enhance understanding of T2DM susceptibility. (ii) Taxonomic inconsistency and the necessity for functional metagenomics: Variations in GM composition among different ethnic groups are frequently inconsistent and may be primarily influenced by dietary habits, medication usage, and environmental factors, rather than by stable taxonomic signatures. In particular, widespread metformin use across T2DM cohorts represents a major pharmacological confounder. Disentangling the independent effects of medication, ethnicity, diet, and disease pathology will require studies of drug-naïve cohorts or rigorous statistical adjustment for pharmacological exposures. Future investigations should prioritize the functional characterization of microbial pathways and metabolites that are pertinent to T2DM, as taxonomically distinct communities may demonstrate functional redundancy, yielding similar metabolites such as SCFAs, secondary bile acids, indole derivatives, and compounds related to trimethylamine. There is a pressing need for longitudinal, multi-ethnic studies that integrate functional metagenomics, metabolomics, and host phenotyping to uncover conserved microbial functions linked to T2DM across various populations. (iii) Validation shortcomings in epigenetic biomarkers and clocks: Epigenetic biomarkers have not been adequately validated across populations with diverse ancestries. The majority of epigenetic clocks have been created using predominantly European cohorts, which raises concerns regarding calibration bias and diminished predictive accuracy in populations that are underrepresented. Variations in baseline DNA methylation, genetic ancestry, population-specific expression quantitative trait loci (eQTLs), cell-type composition, and tissue specificity may further limit their applicability. To enhance the robustness, fairness, and clinical utility of these tools for predicting T2DM risk, extensive longitudinal multi-ethnic studies are essential for validation, recalibration, and external replication. (iv) Methodological fragmentation in cross-omic integration: The integration at the systems level is still constrained due to the tendency to analyze microbiome, epigenome, transcriptome, metabolome, and exposome data separately. This issue is further exacerbated by insufficient adjustments for significant confounders such as psychosocial stress, mental health, neighborhood environment, and digital lifestyle exposures. Future research should implement standardized analytical frameworks and sophisticated techniques, including causal mediation analysis, Mendelian randomization, and network-based machine learning, to enhance causal inference across multi-omic datasets. Given the limited direct human evidence linking the gut microbiota–epigenome axis to ethnic disparities in metabolic disease susceptibility, future research should prioritize longitudinal, multi-ethnic cohort studies with repeated multi-omic profiling and rigorous causal inference approaches, including causal mediation analysis [113] and, where appropriate, Mendelian randomization [114]. These strategies would help clarify whether this axis causally mediates ethnic differences in disease susceptibility or primarily reflects cumulative environmental and exposomal influences. (v) Translational barriers and fair implementation: Although there have been significant strides in understanding mechanisms, the translation into clinical practice is still restricted, as strategies for prevention and treatment seldom take into account genetic ancestry, environmental factors, microbiome makeup, or epigenetic characteristics. New methodologies, such as digital phenotyping and AI-driven risk assessment, have the potential to enhance personalized risk categorization; however, they necessitate prospective validation and fair implementation to prevent exacerbating health inequalities.

## 8. Conclusions

Ultimately, the disproportionate burden of T2DM among racial and ethnic populations may be understood in the context of complex relationships among environmental exposures, the gut microbiota–host epigenetic axis, and host biology rather than as reflecting solely isolated genetic or lifestyle factors. This Review highlights the importance of systems-level approaches that integrate multi-omics, environmental, and clinical data to better understand interindividual and population-level variability in metabolic disease susceptibility. Future progress will require longitudinal, mechanistic, and integrative studies to further investigate potential causal pathways and evaluate how this knowledge may inform more precise, equitable, and microbiome-informed strategies for risk prediction, prevention, and personalized management of T2DM.

## Figures and Tables

**Table 1:** Emerging microbiome- and epigenome-based strategies for precision prevention and management of type 2 diabetes mellitus.

Approach	Biological Target	Potential Clinical Application	Level of Evidence	Current Limitations	Ref.
GM profiling	Gut microbial composition and functional dysbiosis	Risk stratification and early detection of metabolic dysfunction	Early clinical research/biomarker discovery	Population heterogeneity; lack of standardized pipelines	[35]
Epigenetic biomarkers	DNA methylation, histone modifications, ncRNAs	Early diagnosis and individualized risk prediction	Clinical observational/early validation	Limited validation across ethnic populations	[101]
Precision nutrition	GM composition and microbial metabolites	Personalized dietary interventions to improve glycemic control	Emerging clinical application	High interindividual variability	[106]
Prebiotics, probiotics and postbiotics	Modulation of microbial ecology and metabolite production	Adjunctive therapy for T2DM management	Variable clinical trial evidence	Variable efficacy and lack of standardized formulations	[112]
Fecal microbiota transplantation (FMT)	Restoration of gut microbial communities	Improvement of insulin sensitivity in selected individuals	Proof-of-concept clinical trials	Transient effects, durability and safety remain uncertain	[109,110]
Emerging epigenetic therapies	HDACs, DNMTs and other epigenetic regulators	Precision metabolic therapies	Mostly preclinical	Mostly preclinical; limited clinical evidence	[101,102]

## Data Availability

No new data were created or analyzed in this study. Data sharing is not applicable to this article.

## Abbreviations

ABCG1	ATP Binding Cassette Subfamily G Member 1
BCAA	Branched-Chain Amino Acids
BMI	Body mass index
ceRNA	Competing endogenous RNA
CLDN1	Claudin-1
DNAmet	DNA methylation
DNMTs	DNA methyltransferases
DOHaD	Developmental origins of health and disease
EWAS	Epigenome-Wide Association Study
FMT	Fecal Microbiota Transplantation
FXR	Farnesoid X Receptor
GAS5	lncRNA growth arrest-specific 5
GLP-1	Glucagon-like peptide-1
GM	Gut microbiome
GPCR	G-Protein-Coupled Receptor
HDACs	Histone deacetylases
HPA	Hypothalamic–pituitary–adrenal
IDF	International Diabetes Federation
JNK	c-Jun N-terminal kinase
lncRNA	Long Non-Coding RNA
LPS	Lipopolysaccharide
MALAT1	Metastasis Associated Lung Adenocarcinoma Transcript 1
MASLD	Metabolic dysfunction-associated steatotic liver disease
miRNAs	microRNAs
NAFLD	nonalcoholic fatty liver disease
ncRNAs	non-coding RNAs
OCLN	Occludin
PPARGC1A	Peroxisome proliferator-activated receptor gamma coactivator-1α
SAM	S-adenosylmethionine
SCFAs	Short-chain fatty acids
SDOH	Social determinants of health
SOCS3	Suppressor of cytokine signaling 3
T2DM	Type 2 diabetes mellitus
TCF7L2	Transcription factor 7-like 2
TJP1	Tight junction protein 1
TMAO	Trimethylamine-N-oxide
TNF-α	Tumor necrosis factor alpha
TXNIP	Thioredoxin-interacting protein gene
